# Research trends of radiation induced temporal lobe injury in patients with nasopharyngeal carcinoma from 2000 to 2022: a bibliometric analysis

**DOI:** 10.1186/s13014-023-02345-x

**Published:** 2023-09-13

**Authors:** Ying Guan, Bin-Bin Yu, Shuai Liu, Han-Ying Luo, Shi-Ting Huang

**Affiliations:** 1 Department of Radiation Oncology, Guangxi Medical University Cancer Hospital, No 71, Hedi Road, Nanning, 530021 Guangxi People’s Republic of China; 2 Department of Radiotherapy Oncology, The Sixth Affiliated Hospital of Sun Yat-Sen University, Guangzhou, 510655 People’s Republic of China

**Keywords:** Temporal lobe injury, Nasopharyngeal carcinoma, Radiation induced complication, Bibliometric analysis

## Abstract

**Background:**

In patients with nasopharyngeal cancer (NPC), radiation-induced temporal lobe injury (TLI) is the most dreaded late-stage complication following radiation therapy (RT). We currently lack a definitive algorithmic administration for this entity. In the meantime, the pathogenesis of TLI and the mechanism-based interventions to prevent or treat this adverse effect remain unknown. To better answer the aforementioned questions, it is necessary to comprehend the intellectual foundations and prospective trends of this field through bibliometric analysis.

**Methods:**

Articles were gathered from the Web of Science Core Collection (WoSCC) database between 2000 and 2022. CiteSpace was utilized to create a country/institutional co-authorship network, perform dual-map analysis, and find keywords with citation bursts. VOSviewer was used to build networks based on author co-authorship, journal citation, co-citation analysis of authors, references, and journals, and keyword co-occurrence.

**Results:**

A total of 140 articles and reviews were included in the final analysis. The number of publications has steadily increased with some fluctuations over the years. The country and institution contributing most to this field are the China and Sun Yat-Sen University. Han Fei was the most prolific author, while Lee Awm was the most frequently cited. The analysis of co-occurrence revealed three clusters, including: “radiation-induced injury or necrosis in NPC,” “clinical studies on chemotherapy/radiotherapy complications and survival in recurrent NPC,” and “IMRT/chemotherapy outcomes and toxicities in head and neck cancer”). Most recent keyword bursts were “volume,” “temporal lobe injury,” “toxicities,” “model,” “survival,” “intensity modulated radiotherapy,” “induced brain injury,” “head and neck cancer,” and “temporal lobe.”

**Conclusion:**

This study provides some insights of the major areas of interest in the field of radiation-induced TLI in patients with NPC by bibliometric analyses. This study assists scholars in locating collaborators and significant literature in this field, provides guidance for publishing journals, and identifies research hotspots. This analysis acknowledges significant contributions to the discipline and encourages the scientific community to conduct additional research.

## Background

The incidence of nasopharyngeal carcinoma varies widely by region, with a high incidence of more than 30 per 100,000 people in southern China and Southeast Asia and a low incidence of about one per 100,000 people worldwide [1, 2]. Radiation therapy (RT) is the primary modality of treatment for NPC. Gross tumour volume (GTV) must receive a definitive dosage of radiation between 66 and 70 Gy, while clinical target volume (CTV) must receive 54–60 Gy. More than 70% of NPC patients manifest with stage III or stage IV disease, with extensive skull base invasion or even cavernous sinus involvement occurring frequently [3]. Under these conditions, treatment with RT exposes portions of the temporal lobes to dosages greater than 60 Gy. This dramatically increases the likelihood of temporal lobe injury (TLI), one of the most dreaded late-stage complications following radiotherapy in NPC. Even at a late stage, the majority of patients with TLI were asymptomatic [4], whereas 31% of patients with temporal lobe necrosis (TLN) presented with classical temporal lobe epilepsy and 14% with non-specific clinical symptoms including epilepsy, dysphasia, cognitive decline, change in consciousness, memory impairment, dizziness, and headache [4–6]. Radiation-induced TLI may also result in fatal symptoms such as convulsions, intracranial hemorrhage, and herniation [7]. TLI accounts for approximately 65% of fatalities from radiation-induced complications and was associated with a severe reduction in survivors' quality of life (QOL) in NPC patients who received conventional two-dimensional radiotherapy (2D-CRT) [8].

The incidence of individuals with NPC who experienced radiation-induced TLI was influenced by the RT methods and dose. The majority of RT modality for NPC has been intensity-modulated radiotherapy (IMRT) in recent years. The incidence of radiation-induced TLI decreased for patients receiving IMRT, falling between 4.6 and 8.5%, compared to 4.6 to 35% for those receiving traditional two-dimensional conventional radiotherapy (2D-CRT) [9–13].

The increased interest in this subject can be attributed to the fact that, up until now, the study of this side effect has been able to reduce its incidence and there is no definitive algorithmic management for this entity. In the meantime, the pathogenesis of TLI and the mechanism-based interventions to prevent or treat this adverse effect remain unknown. To better answer the aforementioned concerns, it is necessary to comprehend the intellectual foundations and future developments of this field. Consequently, the purpose of this bibliometric analysis was to map the knowledge landscape regarding the underlying pathogenesis and treatment of radiation-induced TLI in NPC patients between 2000 and 2022 in order to better comprehend the evolution and reveal the overall trends, dynamics, and scientific outputs of radiation-induced TLI research, as well as to provide direction for future research.

## Material and methods

### Data source search strategy

All data were obtained from the Web of Science Core Collection (WoSCC) database on July 13, 2022.The medical subject headings (Mesh) and entry terms were used as search strategies. The search strategy was TS = ("Nasopharyngeal Carcinoma" OR "Carcinoma, Nasopharyngeal" OR "Carcinomas, Nasopharyngeal" OR "Nasopharyngeal Carcinomas") AND TS = ("temporal lobe injur*" OR "radiation induced temporal lobe injur*" OR "temporal lobe necrosis" OR "radiation induced temporal lobe necrosis" OR "TLI" OR "TLN" OR "radiation induced complication*"). The timeframe considered for English-language publications was from 2000 to 2022. Two authors (HYL and STH) independently conducted the search and evaluated the articles. Discrepancies were resolved through discussion or consultation with a third author (BBY). The complete bibliographic information for 140 documents was downloaded and imported into CiteSpace (version 6.1.R4, Drexel University, Chaomei Chen). One hundred and forty recordings were identified automatically by the software. No duplicates were located. Finally, 132 articles and eight reviews were incorporated into the study. No ethical approval was required for this investigation because no human subjects were used.

### Data collection

Author names, nationalities and affiliations, article title, year of publication, name of publishing journal, keywords, and abstract were collected from eligible articles. The WoSCC was used to download all records as a.txt file.

### Bibliometric analysis

GraphPad Prism software (version 9.5.1[528], GraphPad Software, San Diego, California USA) was utilized to analyze the publication trend and develop a polynomial regression model Y = B0 + B1*(2023 − MeanX) + B2*((2023 − MeanX)^2) + B3*((2023 − MeanX)^3) to estimate the amount of articles published in 2023.

CiteSpace (version 6.1.R4, Drexel University, Chaomei Chen) is an excellent option for bibliometric analysis of the literature [14, 15]. CiteSpace was used to execute the co-authorship network of nations/institutions, dual-map analysis, and keyword detection with citation bursts. A citation explosion is a crucial indicator for detecting new trends, as it indicates a period of increased interest in the underlying work. CiteSpace parameters included time period (2000–2022), year of slice (1), selection criteria (Top 50), link retaining factor (LRF = 3), look back years (LBY = 5), and e for top N (e = 1).

VOSviewer (version 1.6.18, Leiden University, van EckNJ) was used [16, 17] to construct a network based on co-authorship, journal citation, co-citation analysis of authors, references, and journals, and keyword co-occurrence. Keywords that occurred more than twenty times were also included in the co-occurrence network analysis in order to identify significant terms in the research on radiation-induced TLI in NPC patients.

## Results

### Publication outputs and time trend

**Fig. 1:**
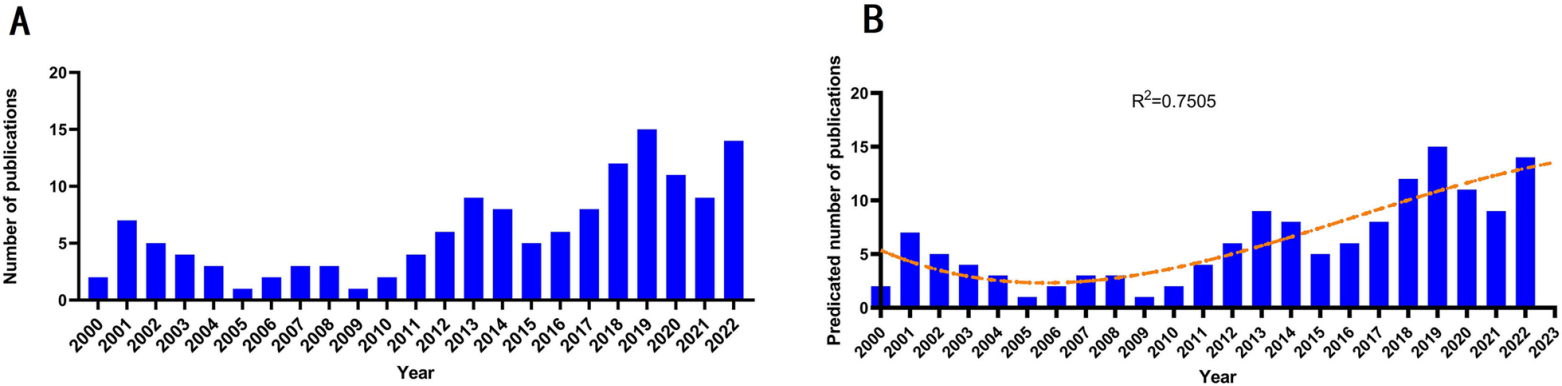
Global trends in publications about TLN in patients with NPC. **A** The single-year publication numbers over the past 20 years. **B** Model fitting curves of growth trends in publications and prediction of future publication numbers

The number of publications on radiation-induced TLI in patients with NPC has climbed continuously over the past 20 years, with occasional oscillations between years, from 2 articles in 2000 to 14 articles in 2022 (Fig. 1A). The time polynomial curve fitting of the number of articles that can forecast future trends was done using the nonlinear regression model (coefficient of determination (R2) = 0.7505, Fig. 1B). This time curve implies that the field is currently experiencing a period of consistent expansion in the number of publications produced globally. Additionally, the number of articles was predicted to reach 13 in 2023 by curve fitting.

### Distribution of country and institution

**Fig. 2:**
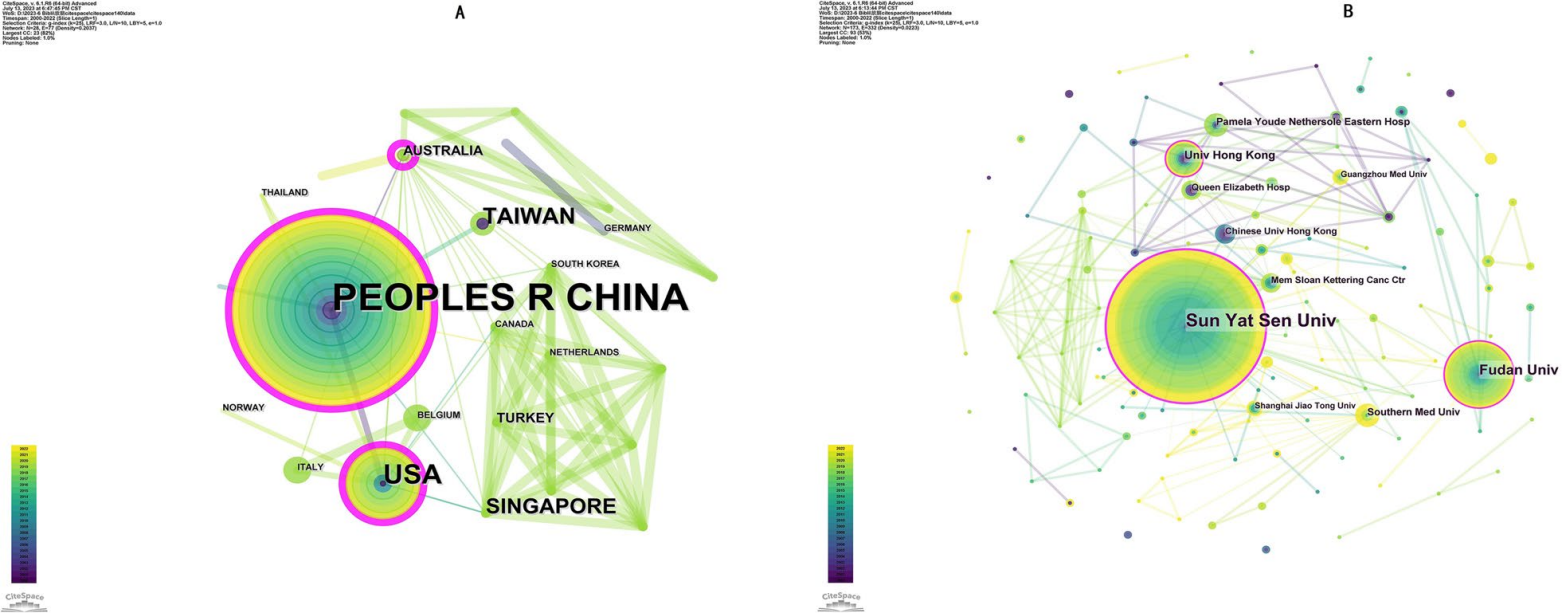
**A** A network map of co-authorship between countries/territories engaged in TLN in patients with NPC. **B** A network map of co-authorship between institutions. In the network map, a point represents a country/territory/institution and a line between 2 points represents the cooperation relationship. A wider line indicates a stronger relationship

CiteSpace analysed the contributions of 56 countries and regions to publications on radiation-induced TLI in patients with NPC (Fig. 2A). China (101 papers), the United States (USA, 27 papers), the Taiwan region (13 papers), Singapore (ten papers), and Australia (four papers) were the top five countries and regions in terms of productivity. Australia (0.65), China (0.28), and England (0.21) were the top three nations in terms of centrality (purple round).

The 485 institutions that contributed to this field were examined using CiteSpace (Fig. 2B). The five most productive institutions were Sun Yat-Sen University (46 papers), Fudan University (17 papers), University of Hong Kong (9 papers), Southern Medical University (6 papers), and Pamela Youde Nethersole Eastern Hospital (6 papers). Sun Yat-Sen University (0.40), Fudan University (0.12), University of Hong Kong (0.11), and Memorial Sloan-Kettering Cancer Centre (0.10) were the four most central institutions (purple round).

### Distribution of journal and research area

A total of 140 articles were published in 65 journals. Table 1 shows the top 10 most popular journals for publishing articles on radiation-induced TLI in patients with NPC. The *International Journal of Radiation Oncology Biology Physics* had the largest number of published articles (18 records,12.86% of all articles), followed by *Radiotherapy and Oncology* (10 records, 7.14%), *Head and Neck-Journal for Sciences and Specialties of the Head and Neck* (10 records, 7.14%), *Radiation Oncology* (7 records, 5.00%), and *Frontiers in Oncology* (5 records, 3.57%).

**Table 1:** The top 5 popular journals and cited journals

Rank	Top 5 popular journals	Documents (n)	Top 5 cited journals	Citations (n)
1	International Journal of Radiation Oncology Biology Physics	18	International Journal of Radiation Oncology Biology Physics	1083
2	Radiotherapy and Oncology	10	Radiotherapy and Oncology	287
3	Head and Neck-Journal for Sciences and Specialties of the Head and Neck	10	Journal of Clinical Oncology	173
4	Radiation Oncology	7	Radiology	128
5	Frontiers in Oncology	5	Head and Neck-Journal for Sciences and Specialties of the Head and Neck	125

**Fig. 3:**
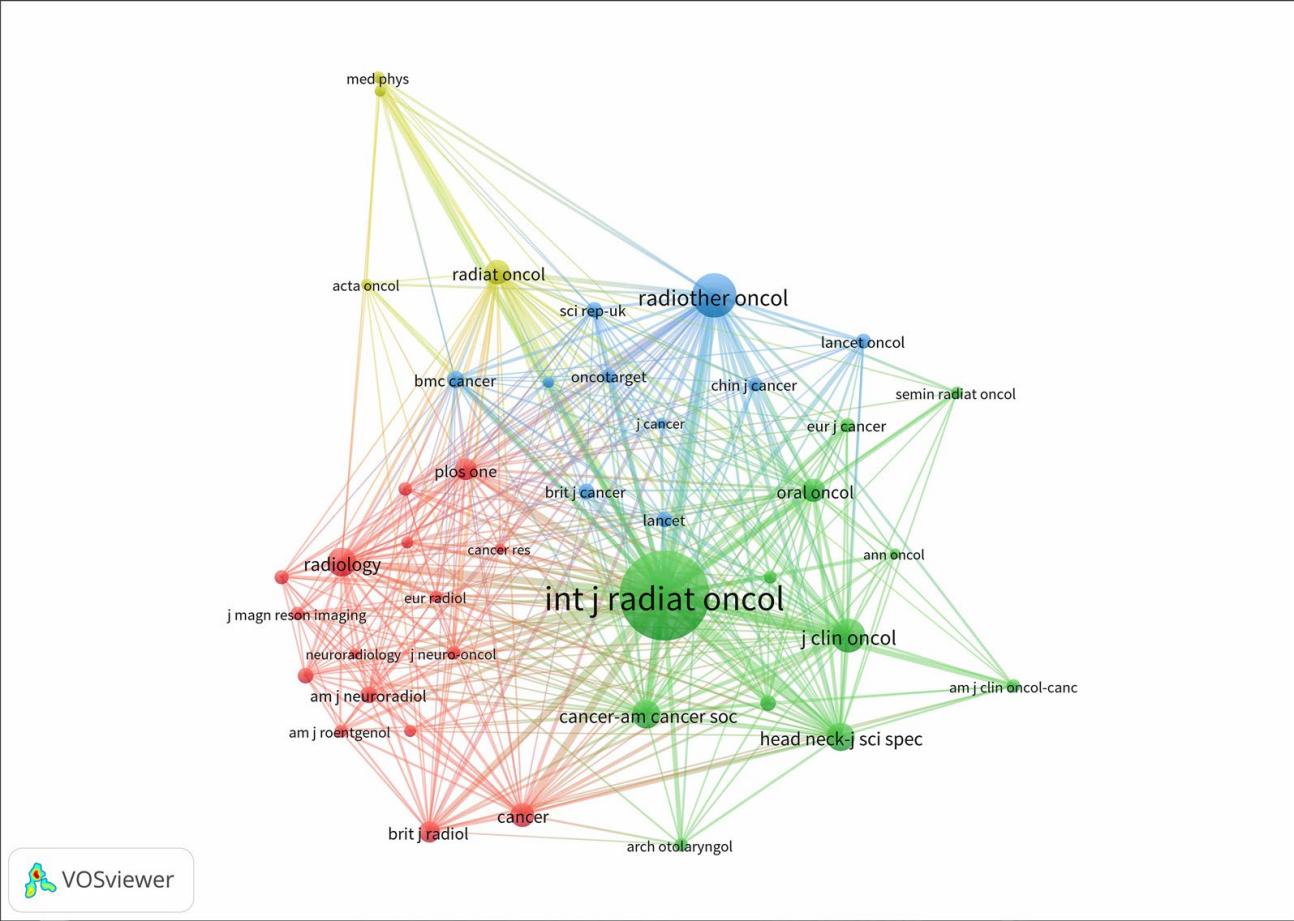
A network map of co-cited journals with more than 20 publications

A total of 42 journals of the references for all publications that were co-cited in more than 20 publications were analyzed by VOSviewer (Fig. 3). The top 5 cited journals were shown in Table 1. *International Journal of Radiation Oncology Biology Physics* had the largest number of citations (1083 citations), followed by *Radiotherapy and Oncology* (287 citations), *Journal of Clinical Oncology* (173 citations), *Radiology* (128 citations), and *Head and Neck-Journal for Sciences and Specialties of the Head and Neck* (125 citations).

**Fig. 4:**
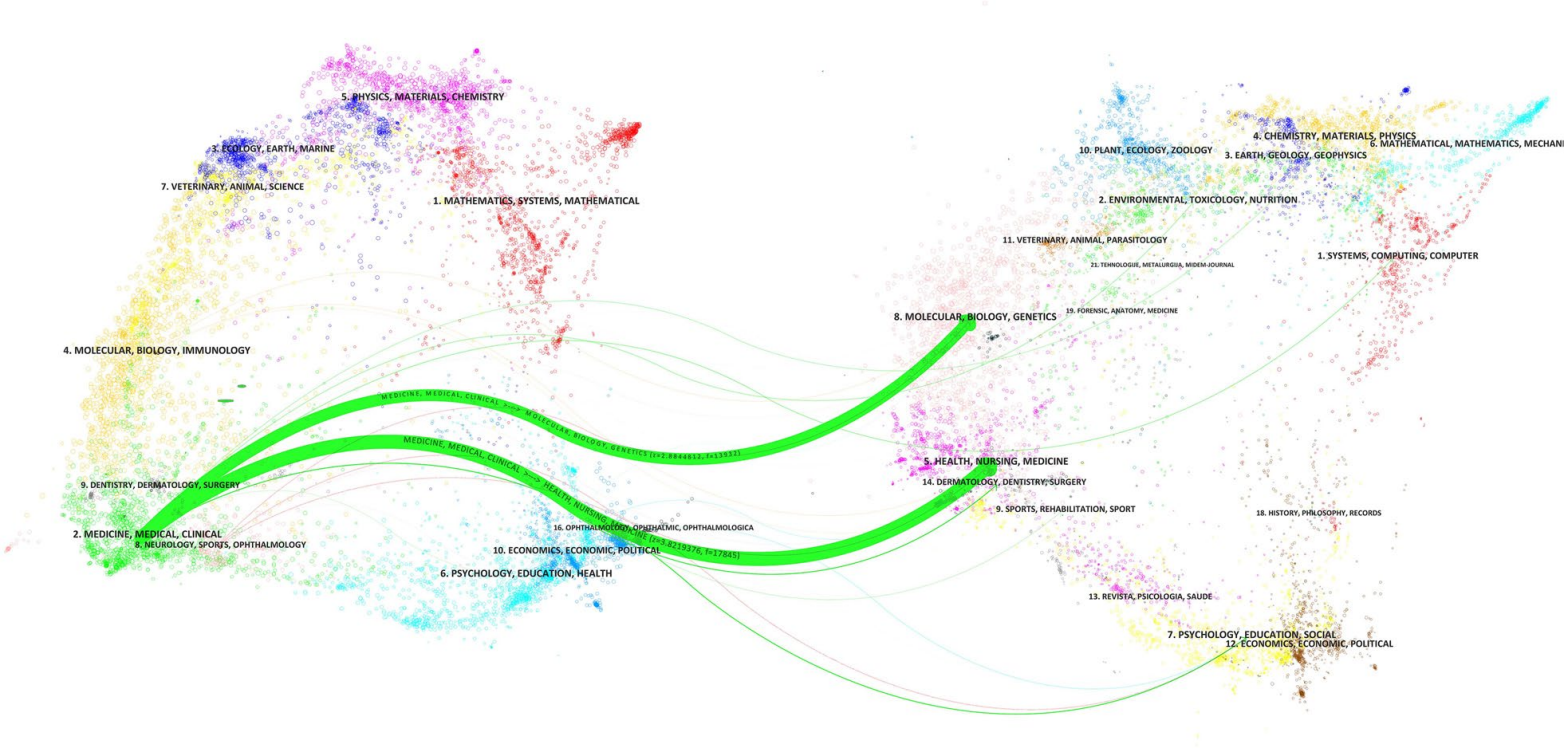
A dual-map overlay of journals related to TLN in patients with NPC

CiteSpace developed a dual-map overlay with citation and cited journal matrices. The majority of papers were published in the field on the left ("Medicine, Medical and Clinical"), which was primarily influenced by the field on the right ("Molecular, Biology, Genetics", z = 2.88 and "Health, Nursing, Medicine", z = 3.82). Figure 4 depicts two primary citation pathways represented as green curves with journal subjects labelled.

The current analysis revealed 19 research areas in total. Table 2 lists the top five most well-represented research areas. Oncology had the highest number of records (87, accounting for 62.14% of all articles), followed by Radiology, Nuclear Medicine Imaging (58, 41.43%), Surgery (15, 10.71%), Otorhinolaryngology (13, 9.29%), and Clinical Neurology (11, 7.86%).

**Table 2:** The top 5 well-represented research areas

	Top 5 research areas	Count	% (of 140)
1	Oncology	87	62.14
2	Radiology, Nuclear Medicine Imaging	58	41.43
3	Surgery	15	10.71
4	Otorhinolaryngology	13	9.29
5	Clinical Neurology	11	7.86

### Analysis of authors

**Fig. 5:**
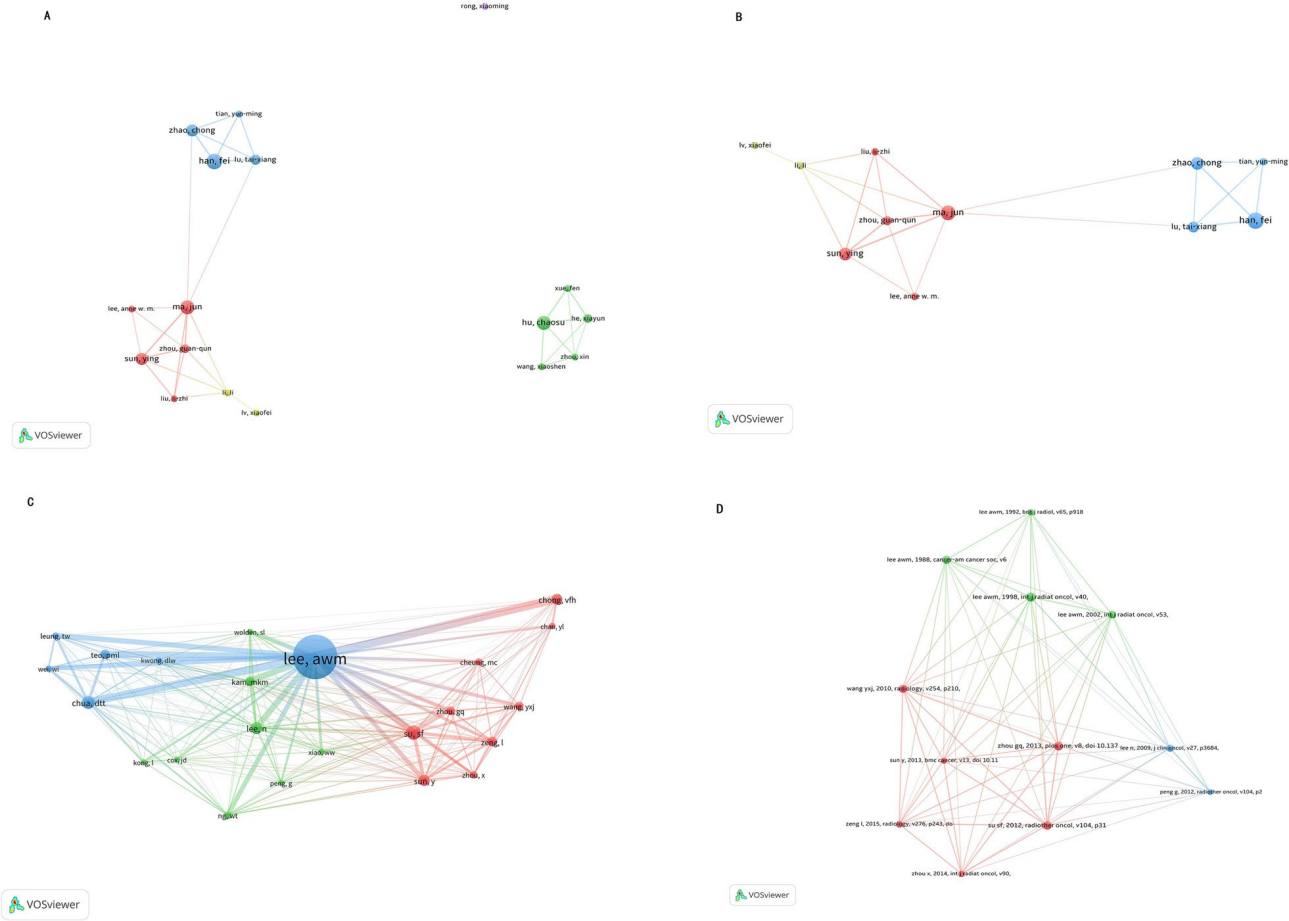
**A** A network map of co-authorship between authors with more than 4 publications. **B** Co-authorship between 11 authors that were connected to each other. Dots represented authors and larger dot indicated a higher number of publications, the links represented author collaborations. **C** A network map of co-citation between authors with more than 20 publications. **D** A network map of co-citation between references with more than 20 publications

VOSviewer examined 17 authors in total who co-authored more than 4 publications (Fig. 5A). Table 3 displayed the top ten most active authors. First place went to Han Fei (9 documents), then Ma Jun (8), Hu Chao-su (8), Sun Ying (7), Zhao Chong (7), Lu Tai-xiang (6), Zhou Guan-qun (6), He Xia-yun (5), Liu Li-zhi (4), and Tian Yun-ming (4). The collaborations of 11 authors were displayed in Fig. 5B after 6 items that weren't related to one another were excluded.

**Table 3:** The top 10 active authors, co-cited authors and co-cited references

Rank	Top 5 active authors	Documents (n)	Top 5 co-cited authors	Citations (n)	Co-cited references	Citation (n)	Type	IF (2022)
1	Han Fei	9	Lee Awm	252	Lee Awm, 1998, int j radiat oncol, v40, p35, https://doi.org/10.1016/s0360-3016(97)00580-4	33	Clinical investigation	7.0
2	Ma Jun	8	Su SF	60	Zhou GQ, 2013, plos one, v8, https://doi.org/10.1371/journal.pone.0067488	32	Clinical investigation	3.752
3	Hu Chaosu	8	Chua Dtt	54	Su SF, 2012, radiother oncol, v104, p312, https://doi.org/10.1016/j.radonc.2012.06.012	31	Clinical investigation	5.7
4	Sun Ying	7	Lee N	48	Wang Yxj, 2010, radiology, v254, p210, https://doi.org/10.1148/radiol.09090428	30	Clinical investigation	29.146
5	Zhao Chong	7	Sun Ying	43	Lee Awm, 1988, v61, p1535, https://doi.org/10.1002/1097-0142(198804 cancer-am cancer soc 15)61:8<1535::aid-cncr2820610809>3.0.co;2-e	30	Clinical investigation	6.2
6	Lu, Tai-xiang	6	Chong, Vfh	42	Lee Awm, 2002, int j radiat oncol, v53, p75, https://doi.org/10.1016/s0360-3016(02)02711-6	29	Clinical investigation	7.0
7	Zhou, Guan-qun	5	Zeng, L	37	Lee n, 2009, j clin oncol, v27, p3684, https://doi.org/10.1200/jco.2008.19.9109	26	Clinical trial	45.3
8	He, Xiayun	5	Kam, Mkm	36	Zeng l, 2015, radiology, v276, p243, https://doi.org/10.1148/radiol.14141721	25	Clinical investigation	29.146
9	Liu, Li-zhi	4	Teo, Pml	36	Zhou X, 2014, int j radiat oncol, v90, p261, https://doi.org/10.1016/j.ijrobp.2014.05.036	23	Clinical investigation	7.0
10	Tian, Yun-ming	4	Zhou, Guan-qun	32	Lee Awm, 1992, brit j radiol, v65, p918, https://doi.org/10.1259/0007-1285-65-778-918	23	Clinical investigation	3.629

*IF* impact factors

Twenty-three authors and twelve references that were co-cited in more than 20 publications were analysed using VOSviewer by data from all publications included in this study (Fig. 5C, D). Table 3 displays the top 10 most-cited authors. Lee Awm had the most citations (252), followed by Su SF (60), Chua Dtt (54), Lee N (48), Sun Y (43), Chong Vfh (42), Zeng L (37), Kam Mkm (36), Teo Pml (36), and Zhou Guan-qun (32).

Table 3 also summarizes the top 10 co-cited references. Among them, the paper entitled “Effect of time, dose, and fraction on temporal lobe necrosis following radiotherapy for nasopharyngeal carcinoma” published by Lee Awm in *International Journal of Radiation Oncology Biology Physics* had the most co-citations (n = 33).

### Analysis of keywords

**Fig. 6:**
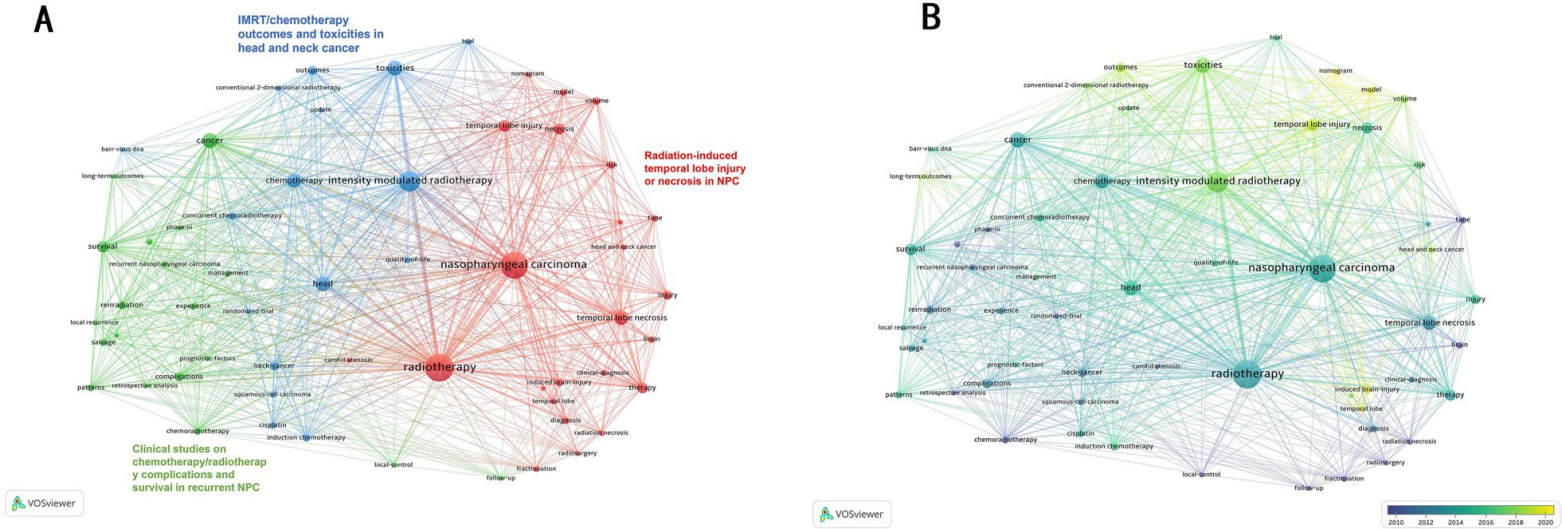
Analysis of keywords. **A** Mapping of keywords of studies (categorized into 3 clusters). **B** Distribution of keywords according to average publication year (blue: earlier, yellow: later)

VOSviewer was used to conduct an analysis of the keywords contained within the 140 publications that were taken into consideration for this study (Fig. 6A). A total of 59 different keywords were found to have occurred five times or more throughout the research. The VOSviewer was used to assign a color code to each of the discovered keywords based on the average publication year (Fig. 6B). There were three major clusters whose research directions were different but correlated. “Radiotherapy (100),” “nasopharyngeal carcinoma (91)”, “temporal lobe necrosis (30)”,“temporal lobe injury (22)”, and “therapy (18)” were the top five keywords with the highest frequency of occurrence in the red cluster, as were “cancer (33),” “survival (21),” “complications (14),” “reirradiation (12),” and “pattens (9)” in the green cluster, and “intensity modulated radiotherapy (60),” “toxicities (34),” “head (32),” “chemotherapy (31),” and “neck-cancer (14)” in the blue cluster. These clusters reflected three main topics of radiation-induced TLI: “radiation-induced temporal lobe injury or necrosis in NPC” “clinical studies on chemotherapy/radiotherapy complications and survival in recurrent NPC,” and “IMRT/chemotherapy outcomes and toxicities in head and neck cancer.”

CiteSpace was utilized to search for burst keywords, which were then determined to be indicative of developing trends (Table 4). After 2017, the keywords that saw the greatest increase in the number of citations in this field were: “volume (strength = 1.69),” “temporal lobe injury (4.29),” “toxicities (4.72),” “model (3.34),” “survival (2.91),” “intensity modulated radiotherapy (1.78),” “induced brain injury (2.7),” “head and neck cancer (1.71),” and “temporal lobe (2.35).”

**Table 4:**
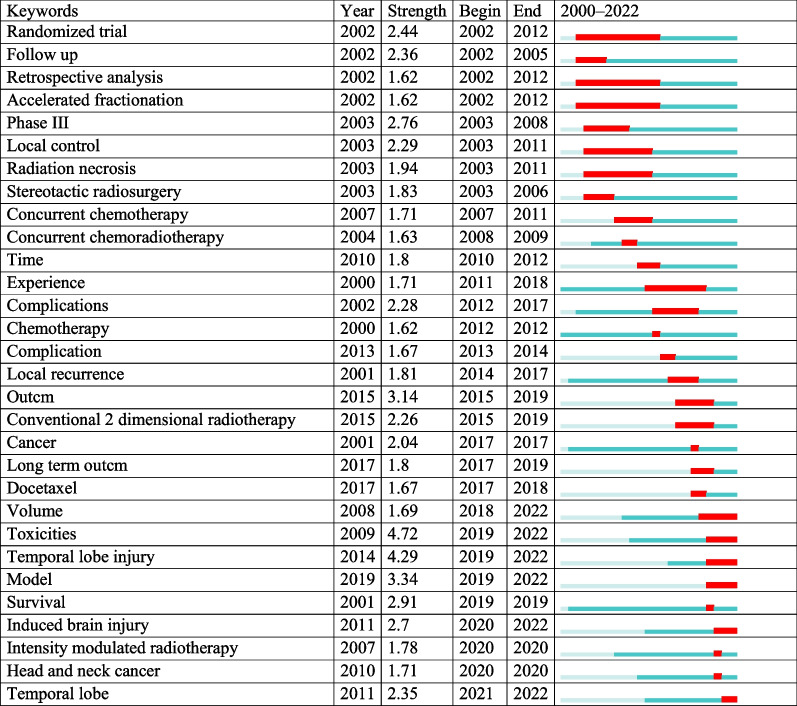
Top 30 keywords with the strongest citation bursts sorted by the beginning year

The red bars mean the keywords occurred frequently; the blue bars mean the keywords occurred infrequently. A greater strength indicates a higher frequency of occurrence

## Discussion

In this study, we applied bibliometric methods for the first time to reveal the intellectual bases and emerging trends of radiation-induced TLI in patients with NPC, aiming to inform readers of the recent knowledge and future research directions of this field. In addition, we also introduced journals, institutions, and authors that readers could rely on to obtain knowledge on this subject.

### General trends in the research field from 2000 to 2022

Radiation-induced TLI in patients with NPC has received a growing amount of attention. The total quantity of publications has steadily increased over the past 2 decades. In addition, it is anticipated that the annual publication output will increase significantly over the next decade, which bodes well for the future of this research field. China, the United States, Taiwan region, Singapore, and Australia all made significant contributions to the publication of articles. China, the United States, and Australia collaborated with numerous nations in this field. Top 5 productive institutions accounted for 84 articles. Among them, the Sun Yat-sen University ranked first, followed by the University of Hong Kong and Southern Medical University. The Sun Yat-sen University had the highest centrality (0.12), indicating that it collaborated closely with numerous institutions. Analyses of co-authorship by country/region and institution indicate that international cooperation is becoming more prevalent in this field.

Top five performing journals published fifty articles, making up 35.71% of all articles. These journals' impact factors (IF) in 2022 range from 2.90 to 7. The *International Journal of Radiation Oncology Biology Physics* (IF, 2022 = 7), *Radiotherapy and Oncology* in Cancer (IF, 2022 = 5.7), known in the field relating to radiation oncology as the Red Journal and Green Journal, and Radiation Oncology (IF, 2022 = 3.6) focus on clinical radiotherapy, combined modality treatment, translational studies, epidemiological outcomes, imaging, dosimetry, and radiation therapy planning, experimental work in radiobiology, chemobiology, hyperthermia and tumour biology, as well as data science in radiation oncology and physics aspects relevant to oncology. *Head and Neck-Journal for Sciences and Specialties of the Head and Neck* was multidisciplinary journal within a scope in the diagnosis and management of diseases of the head and neck. The last one journal highlights research in carcinogenesis and tumor progression, bridging the gap between basic research and clinical applications to advance diagnosis, personalized therapeutics and management strategies as well.

Additionally, a co-citation analysis identified the highly cited, influential studies in this subject. The American Society for Radiation Oncology (ASTRO) official journal International Journal of Radiation Oncology Biology Physics had the most citations (1083), and it published technical developments related to dosimetry and conformal radiation treatment planning. It also published basic science studies looking at tumour physiology, the molecular biology underlying cancer, and the radiation response of normal tissue. Therefore, it is appropriate to view the initial laboratory and clinical studies on radiation-induced TLI that have been published in this journal as landmark studies. To publish a manuscript in the International Journal of Radiation Oncology Biology Physics will be a significant task for researchers. Oncology has the highest percentage of papers (62.14%), making it the most well-represented research field. Co-authorship and co-citation analyses in writers may show possible collaborators and promising authors in this field.

Zhou Guan-qun and Sun Ying are both among the top ten most active and cited authors. Zhou Guan-qun et al. demonstrated that IMRT provides superior protection for TLs compared to 2D-RT. The actuarial 5-year incidence of TLN was substantially lower in IMRT (16%) than in 2D-RT (34.9%; *p* = 0.001), and IMRT could significantly reduce the risk of TLN in T1–T3 diseases but not in T4 diseases [9]. The research of Sun Ying et al. indicated that D0.5 cc of 69 Gy as the dose tolerance of TLs may be suitable for IMRT and that the risk of TLN was largely dependent on high dose hot spots of TLs [18]. The red cluster on the co-authorship analysis network map suggested that Sun Ying et al. from the Sun Yat-Sen University Cancer Centre work with Lee Awm from the University of Hong Kong. The blue cluster showed the collaboration between Lu Tai-xiang et al. from the Sun Yat-Sen University Cancer Center and Tian Yun-ming from the Huizhou hospital. The yellow cluster indicated that Li li and Lv Xiao-fei from the Sun Yat-sen University Cancer Centre collaborate.

The foundational works in this field were the co-cited references. The first place goes to Lee Awm's article "Effect of time, dose, and fraction on temporal lobe necrosis following radiotherapy for nasopharyngeal carcinoma" that was published in the *International Journal of Radiation Oncology Biology Physics*. According to Lee Awm et al., the incidence of radiation-induced TLN patients with NPC was influenced by the RT methods and dosage. The actuarial incidence of TLN for NPC patients receiving conventional 2D-RT ranged from 4.6% in a conventional dosage 2.5 Gy per fraction scheme of 60 Gy to 18.6% in an accelerated dose scheme of 4.2 Gy per fraction during a 10-year period. The same research also showed that an increase in dose per fraction could dramatically increase the incidence rate of TLN despite a lower overall dose when considering the effect of dose scheme (fractionation) on TLN [12].

### Keywords as indicators of emerging trends

The majority of studies have focused on radiation-induced TLI in NPC patients treated with IMRT in the last 5 years, according to the keyword co-occurrence atlas and most recent strong citation bursts analysis [19]. This is because more than 70% of NPC patients have locally advanced disease when they are first diagnosed, and the combination of IMRT and chemotherapy is the primary treatment. When it comes to the therapy of TLI, prevention is always preferable to cure. Radiomics, genomics, and other forms of artificial intelligence have recently been used to predict survival outcomes in cancer, including diagnosis, prognosis, therapy response, and radiation-related toxicities or complications. In patients with post-RT NPC, such technologies may be utilized to predict TLI. Thus, the establishment and validation of genomic, radiomics based on magnetic resonance imaging (MRI), normal tissue complication probability (NTCP), risk factors nomograms, and so on prediction models for TLI for this group have just recently become research hotspots.

TLI significant clinical risk factors like chemotherapy and N (node) classification were identified in NPC patients [10, 18]. As for dosimetric parameters, Dmax (maximum point dose, Gy), D1 cc (the dose delivered to the 1 cubic centimeter volume, Gy), and D0.5 cc (the dose delivered to the 0.5 cubic centimeter volume, Gy) were identified as risk factors [9]. Prescription dose and organ constraints are assumed to NTCP models. NTCP models relating dose to the probability of toxicities can guide to make the optimal treatment strategy decision in radiotherapy. TLI was one of the common toxicities were not negligible even with advanced radiotherapy technique and dose strategies in head and neck cancer and central nervous system (CNS) tumor.

For photon-specific data, one study of pediatric stereotactic radiosurgery/stereotactic ablative body radiotherapy (SRS/SABR), the risk of symptomatic brain radionecrosis was associated with an exceeding cumulative dose of 200 Gy biological equivalent dose (BED) by Chandy et al. [20]. On the other hand, Bruni et al. reported that single-session SRS with frameless immobilization head-neck mask tomotherapy was a feasible and safe treatment option for patients with brain metastases, with a good overall response rate and acceptable toxicity. Among toxicities, radiation-related white matter changes (WMC) were detected with MRI in 2/68 patients (3%) and radionecrosis in other 2 patients (3%) [21].

With regards to proton, its physical rationale has supported and justified its use in CNS tumor. Note of intrinsic interpatient variability in radiosensitivity is a radiobiological reality existing no matter how narrow the patient selection criteria. Niemierko et al. found no clear correlation between the risk of brain radionecrosis and elevated linear energy transfer (LET) and thus elevated relative biological effectiveness (RBE). The heterogeneity of LET effect in patients might be small compared with interpatient variability of radiosensitivity. The toxicities could be more affected by interpatient variations [22]. The preliminary clinical outcomes of proton therapy for pediatric, adolescent, and adult CNS tumor treated within the UK National Health Service started the Proton Overseas Programme (POP) are promising. The rate of late effects is comparable to published cohorts according to the. But a significant proportion of grade 3 or higher CNS toxicities were observed in radioresistant tumors (skull base chordoma and chondrosarcomas), which pose challenges due to the high doses (> 70 Gy) required for local control and the proximity of critical structures [23]. A reduction of grade 3 toxicity is clinically relevant and important in justifying the higher cost of proton therapy. In recent years, specific NTCP have been modeling for patient selection for proton therapy [22, 24]. Schröder et al. develop a NTCP model combining clinical and dosimetric factors focused on grade ≥ 2 radionecrosis in a large cohort of patients receiving proton, the best fit was found for the model containing age, prescription dose, D1cc (Gy), and hypertensive blood pressure as risk factors [25]. External validation will be the next step to improve generalizability and potential introduction into clinical routine. Such as randomised and observational studies, can provide clinical and dosimetric data for NTCP modelling, strengthen the existing evidence and aid patient selection.

It is still unclear what causes TLI from its underlying mechanisms. This is owing to the complexity of the CNS, as well as the dose pattern, time course, and feedback mechanisms, making it challenging to model the condition using in vitro and animal systems [26]. Mechanistic investigations are ongoing because the pathophysiology of TLI has not been fully elucidated. Future research will need to focus on more thorough clinical trials and related mechanism studies. Up until this point, conventional therapy, which includes steroids and anticoagulants, has only been effective as palliative care for TLI [7, 27–29]. However, because it has the ability to reverse the underlying pathophysiology, bevacizumab has recently attracted a lot of interest in the care of this entity. Bevacizumab may offer a potential alternative to surgery and steroid therapy, according to studies [28, 30–34]. The molecular pathophysiology of TLI has to be further understood because it may lead to novel therapy possibilities.

### Strengths and limitations

This bibliometric analysis is the first to look at the development of research on radiation-induced TLI in NPC patients. Through investigations of co-authorship, co-citation, cooccurrence, and citation burst, we constructed and visualized the bibliometric networks using 2 well-known scientometric software tools (VOSviewer and CiteSpace). However, this study does have certain shortcomings. First of all, less qualitative analysis is used in this study and more quantitative analysis. Second, the WoSCC database was mostly used for the searches. The outcomes would be improved if they were coupled with data from other databases, such Scopus and PubMed. But it should be highlighted that WoSCC is the most popular scientometrics database.

## Conclusion

This study provides some insights of the major areas of interest in the field of radiation-induced TLI in patients with NPC with bibliometric analyses. It summarizes the progression of the research trend of radiation-induced TLI in the past 20 years. Research publications are increasing year by year in this field. This study assists scholars in locating collaborators and significant literature, provides guidance for publishing journals, and identifies research hotspots. This analysis acknowledges significant contributions to the discipline and encourages the scientific community to conduct additional research.

## Abbreviations

NPC: Nasopharyngeal cancer
TLI: Temporal lobe injury
RT: Radiation therapy
WoSCC: Web of Science Core Collection
GTV: Gross tumour volume
CTV: Clinical target volume
TLN: Temporal lobe necrosis
QOL: Quality of life
2D-CRT: Two-dimensional conventional radiotherapy
IMRT: Intensity-modulated radiotherapy
Mesh: Medical subject headings
USA: United States
MRI: Magnetic resonance imaging
NTCP: Normal tissue complication probability
Dmax: Maximum point dose, Gy
D1 cc: Dose delivered to the 1 cubic centimeter volume, Gy
D0.5cc: Dose delivered to the 0.5 cubic centimeter volume, Gy
CNS: Central nerves system
SRS/SABR: Stereotactic radiosurgery/stereotactic ablative body radiotherapy
BED: Biological equivalent dose
LET: Linear energy transfer
RBE: Relative biological effectiveness
UK: United Kingdom
POP: Proton overseas programme
